# Spatial Characteristics of Double Puncture in Dental Anesthetic Cartridges: A Descriptive Morphological Analysis of Procedural Puncture Patterns

**DOI:** 10.7759/cureus.103079

**Published:** 2026-02-06

**Authors:** Takutoshi Inoue, Toru Yamamoto, Naotaka Kishimoto

**Affiliations:** 1 Department of Anatomy, Teikyo University School of Medicine, Tokyo, JPN; 2 Division of Dental Anesthesiology, Faculty of Dentistry & Graduate School of Medical and Dental Sciences, Niigata University, Niigata, JPN

**Keywords:** cartridge, dental anesthesia, double puncture, local anesthesia, rubber stopper

## Abstract

Introduction

Puncture of the rubber stopper on dental local anesthetic cartridges is routinely performed during dental practice. Repeated puncture at the same site has been suggested to increase the risk of rubber damage, yet no quantitative evaluation of spatial relationships in double puncture has been reported. This study aimed to characterize the spatial features of double puncture using morphological and spatial indices.

Materials and methods

One hundred used dental anesthetic cartridges were examined. The first puncture represented routine clinical practice, whereas the second was performed experimentally using a standardized technique. Puncture sites were imaged under a stereomicroscope, and spatial indices, including circle diameter, inclusion rate, and minimum inter-puncture distance, were measured using image superimposition analysis.

Results

The mean circle diameter was significantly larger at the first puncture than the second (1.59 ± 0.07 vs. 1.31 ± 0.06 mm, p < 0.05). The inclusion rate was 90 ± 3.2%. Minimum distance measurements showed that 33% of cartridges demonstrated inter-puncture proximity of ≤ 0.25 mm.

Conclusion

Double puncture frequently converges within a restricted area. Spatial overlap may concentrate mechanical stress on the stopper and may represent a potential contributing factor to rubber damage. When an initial puncture fails, replacing the cartridge rather than performing a repuncture may be a reasonable precautionary measure.

## Introduction

Local anesthesia is a fundamental procedure in modern dental practice, serving a critical role in pain control and patient comfort during dental treatment [1]. Accordingly, dentists have been reported to use more than 1,500 dental local anesthetic cartridges annually [2].

In Japan, several notable developments in dental local anesthesia have emerged. Most prominently, articaine became commercially available for dental use in January 2025, joining lidocaine, propitocaine, and mepivacaine as the fourth local anesthetic agent approved for routine practice [1]. This approval has facilitated broader access to anesthetic formulations and has been widely utilized internationally.

Dental local anesthetics are supplied in specialized glass cartridges sealed with rubber stoppers, which are punctured using dental needles (27G-33G) during routine preparation. “Coring” refers to the generation of small rubber particles when a needle punctures a rubber stopper, and fragments of stopper material may enter the injectable solution [3,4]. Improper puncture of the rubber stopper has been identified as a potential cause of coring, a phenomenon in which small fragments of rubber detach from the stopper and may contaminate the anesthetic solution [5,6]. Studies have demonstrated that procedural factors such as needle gauge and insertion angle can influence the incidence of coring, with larger-bore needles associated with increased particle generation [7]. Rubber stopper material properties and needle geometry have also been implicated in the likelihood of coring occurrence [8].

In a previous investigation examining the manual dilution of adrenaline within dental cartridges, performed to reduce adrenaline concentration, we reported that such manipulation may necessitate additional puncture of the rubber stopper [5]. Importantly, this dilution procedure is not specified in the manufacturer’s package insert and warrants caution from regulatory and infection-control perspectives. In addition, in clinical settings, unintentional re-puncture of the same cartridge may occur when the initial puncture fails due to incomplete stopper penetration or divergence of the needle tip.

We also previously observed rubber stoppers using scanning electron microscopy and identified precoring (incomplete coring) around puncture sites [6]. Unlike coring, precoring involves partial material detachment that remains adherent at the puncture site. Theoretical progression from precoring to coring may occur if subsequent puncture passes through the compromised region. Notably, however, no studies have quantitatively or spatially evaluated the relative position or overlap of puncture sites when double puncture occurs.

The present study does not directly measure coring but instead investigates spatial puncture relationships as a descriptive, exploratory analysis intended to generate foundational data for future coring-focused research.

The primary objective of this study was to quantitatively characterize the spatial relationship between two puncture sites created in dental local anesthetic cartridges, using coordinate-based assessment.

The secondary objectives were to evaluate, through three spatial indicators: (1) the diameter of the fitted circle at each puncture site, (2) the inclusion rate of first puncture sites inside the second puncture site circle, and (3) the minimum distance between puncture sites, the reproducibility and distribution characteristics of puncture positioning.

This descriptive analysis aims to elucidate spatial puncture patterns during double puncture and provide hypothesis-generating insight into potential mechanical stresses that may contribute to localized rubber stopper damage and potential coring during repeated puncture procedures in clinical practice.

## Materials and methods

Following a previously reported approach [5], this study included 100 dental local anesthetic cartridges (ORA Injection Dental Cartridge 1.8 mL; GC Showa Yakuhin Co., Ltd., Tokyo, Japan) that had been punctured using a 33-gauge dental injection needle (NIProject Dental Needle; NIPRO CORPORATION, Osaka, Japan). All cartridges were derived from the same product lot and were equipped with gray butyl rubber stoppers.

The cartridges were collected immediately following routine clinical administration of local anesthesia and were fully anonymized prior to analysis. The nominal outer diameter of a 33G dental needle commonly used in clinical dentistry is approximately 0.21 mm.

This study did not involve human or animal subjects and utilized only discarded medical materials. Accordingly, institutional ethical review was waived.

Puncture technique

First Puncture (Clinical Condition)

**Figure 1:**
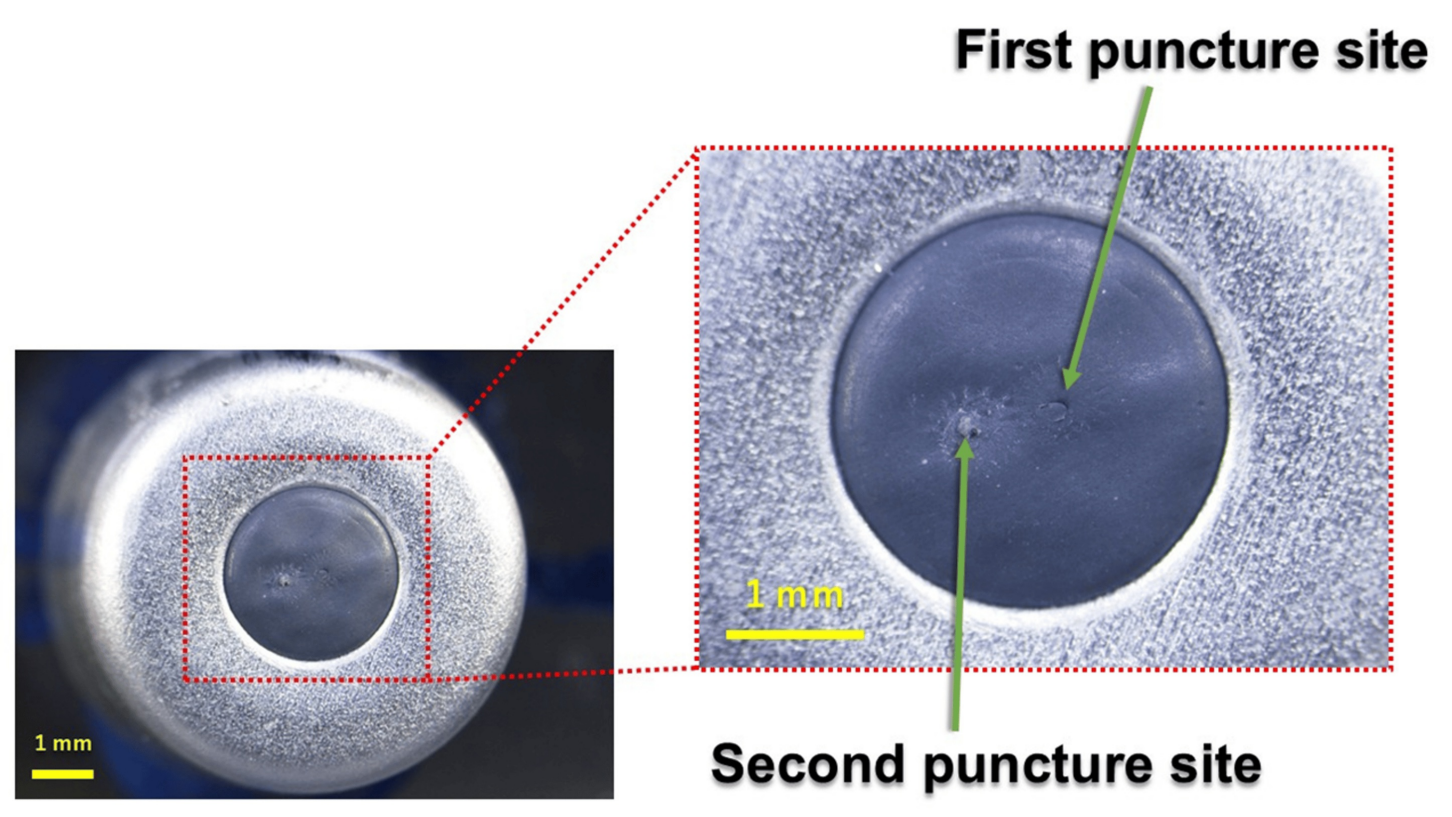
Enlarged view of the cartridge rubber stopper showing the first and second puncture sites

The first puncture corresponded to the initial needle insertion performed in routine clinical settings by dental healthcare personnel (dentists, dental hygienists, or dental assistants) during preparation of the anesthetic cartridge. The analyzed cartridges were subsequently used to administer local anesthesia to patients (Figure 1).

Because this puncture reflected actual clinical operation, parameters such as insertion angle, insertion depth, needle manipulation, and cartridge handling were not standardized and varied among operators.

Second Puncture (Standardized Experimental Condition)

The second puncture was performed experimentally according to the standard procedure specified in the manufacturer’s package insert. Each used cartridge was loaded into a dental syringe (ORA Inj. 1.8D syringe; GC Showa Yakuhin Co., Ltd., Tokyo, Japan) in a fixed orientation, ensuring that the printed registration number on the cartridge surface was positioned consistently to prevent rotational or directional variation.

A small amount of white paint was applied to the tip of a new 33G dental injection needle prior to puncture. The needle was then inserted toward the syringe at a predefined angle and fixed in position along the syringe axis.

All second punctures were performed by a single trained investigator under identical conditions, thereby eliminating operator-dependent variation in insertion angle, penetration trajectory, and handling technique, and ensuring highly reproducible puncture conditions (Figure 1). The needle was inserted approximately perpendicular to the stopper surface (estimated angle ~90° relative to the rubber surface) using a consistent manual technique. Although insertion force was not instrumentally measured, uniform manual pressure was applied across all procedures to minimize procedural variability.

Image acquisition and measurement items

Image Collection

**Figure 2:**
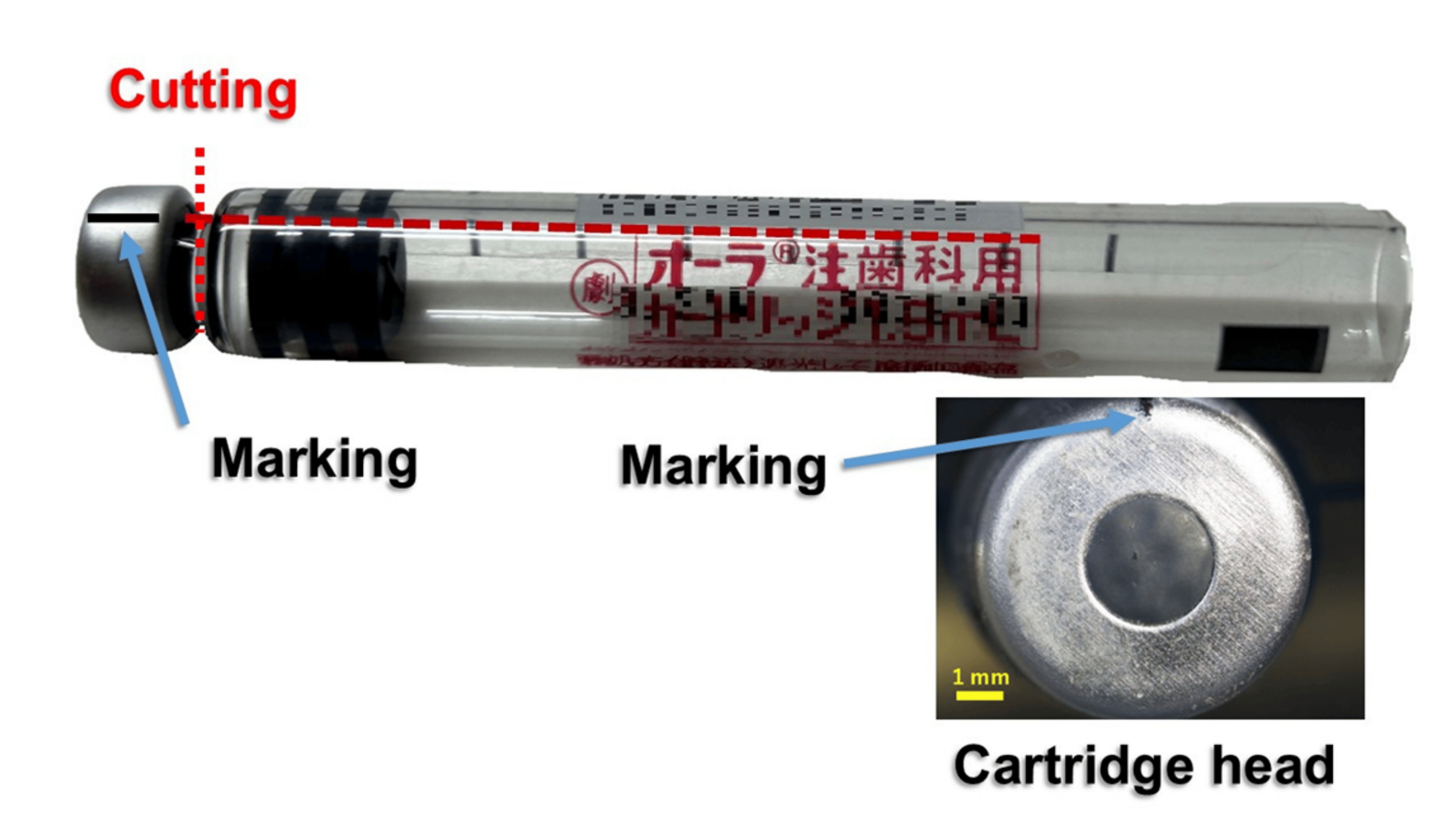
Marking of the cartridge head and sectioning of the cartridge neck Reference marks were placed on the cartridge head using the printed label as a guide to ensure consistent alignment when overlapping the 20 images. After marking, the neck portion of each cartridge was sectioned prior to stereomicroscopic imaging.

Following a previously described protocol [5], a reference mark was applied to each cartridge head, and the neck was sectioned using a dental laboratory burr (Figure 2). The two puncture sites on the rubber stopper were photographed under a stereomicroscope (SZX16; Olympus, Tokyo, Japan).

Image Superimposition and Spatial Analysis

Each puncture site was marked on the acquired images, after which 20 photographs corresponding to each registration number were superimposed using Adobe Photoshop Elements 2025, generating 10 composite images in total.

**Figure 3:**
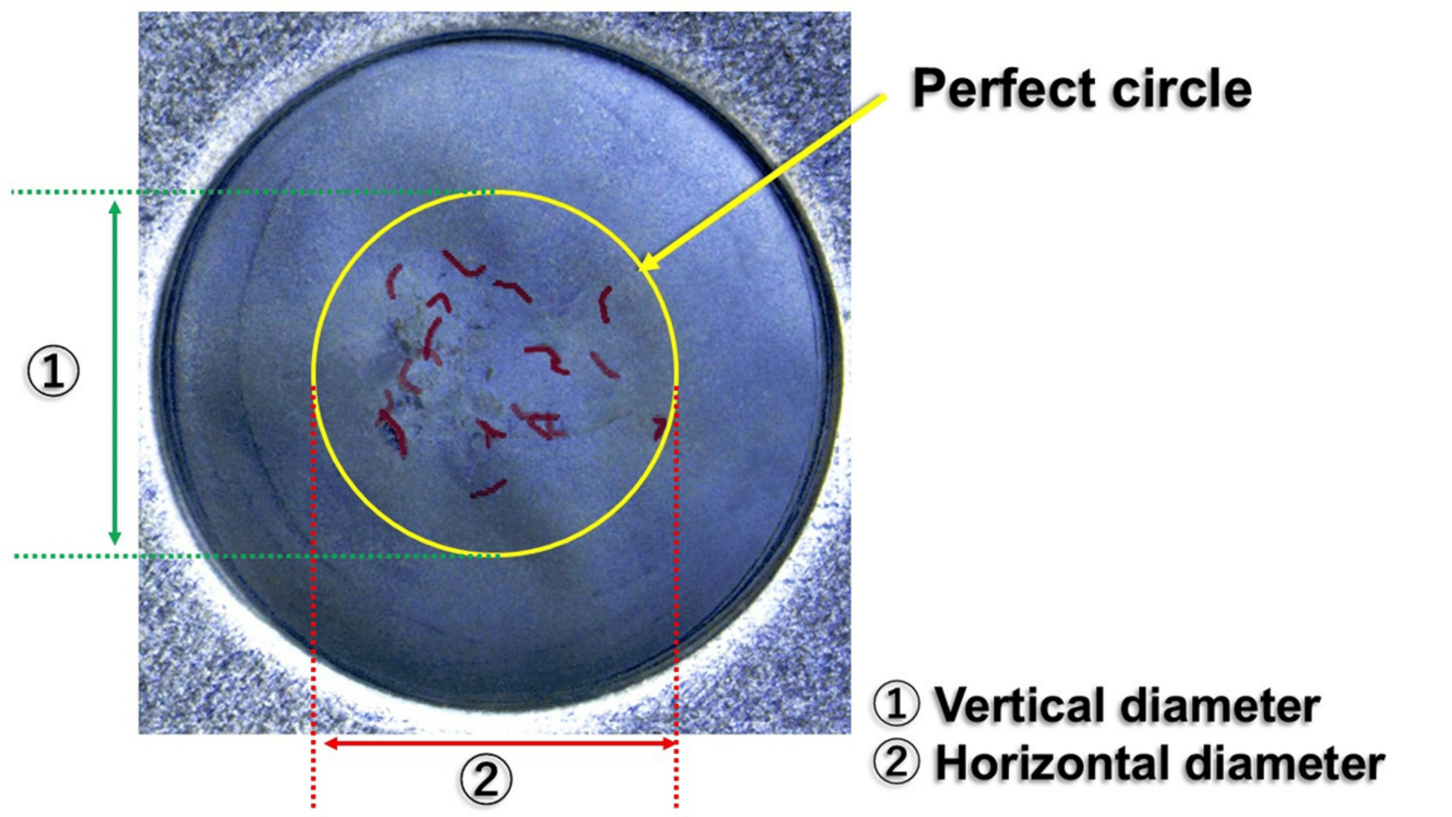
Example of superimposed images and measurement parameters Puncture sites in each of the 20 images were first highlighted. These images were then overlaid to generate a composite view. On the resulting composite image, a circle was inscribed, and its vertical and horizontal diameters were recorded.

On each composite image, the smallest perfect circle encompassing all 20 puncture points was drawn, and its vertical and horizontal diameters (mm) were measured (Figure 3). The mean of these two values was recorded as the circle diameter.

**Figure 4:**
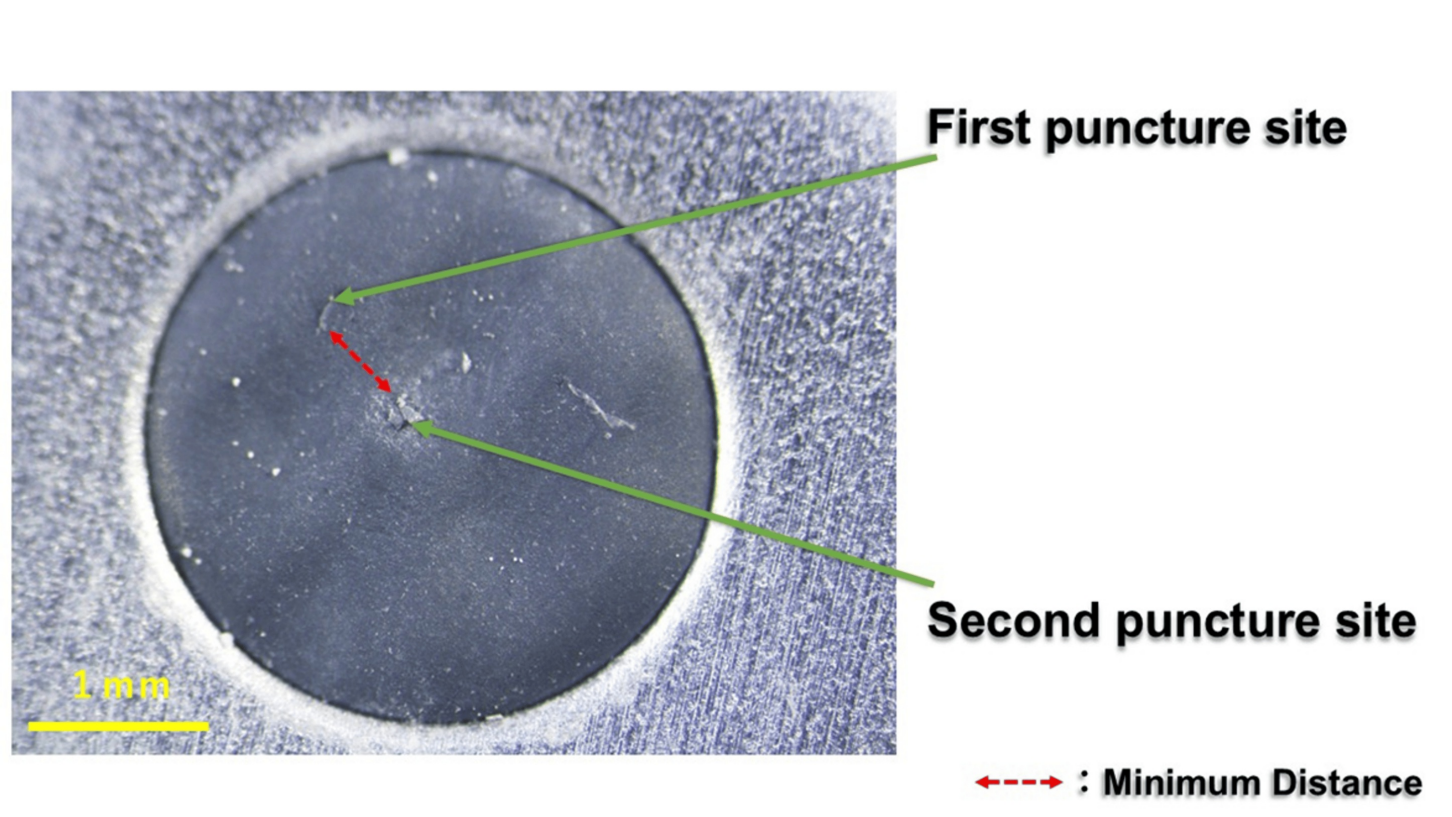
Example of the shortest distance between the two puncture sites The minimum distance (mm) between the first and second puncture sites was measured on each image.

The number of first puncture sites located within the circle derived from the second puncture was counted, yielding the inclusion rate. For all 100 cartridges, the minimum linear distance (mm) between the first and second puncture sites was also measured individually (Figure 4).

These three spatial indices were used descriptively and exploratorily to characterize puncture reproducibility and spatial proximity. These parameters were selected because they allow quantitative representation of puncture dispersion and overlap while remaining measurable from two-dimensional stereomicroscopic images without destructive sample preparation.

Calibration of image measurements was performed using a stage micrometer under the same magnification settings. Pixel dimensions were converted to millimeters using the microscope’s calibrated scale to ensure measurement accuracy.

Rationale for Sample Size

A total of 20 images per registration number were selected based on preliminary pilot evaluation. This number provided an adequate representation of puncture distribution while maintaining image resolution. Because this investigation aimed to descriptively characterize spatial puncture patterns rather than test hypotheses, a priori sample size calculation was not performed.

Statistical analysis

All values were expressed as mean ± standard deviation (SD) or median (interquartile range), as appropriate. Statistical analyses were conducted using Microsoft 365® Excel and EZR (version 1.68; Saitama Medical Center, Jichi Medical University, Saitama, Japan) [9].

Unpaired t-tests were used to compare vertical and horizontal diameters measured from five composite images for the first and second punctures. Paired t-tests were then applied to compare the overall mean diameters between first and second punctures obtained from the five image sets. Homogeneity of variances was assessed using F-tests. A p-value < 0.05 was considered statistically significant.

Minimum inter-puncture distances were treated as continuous variables and summarized descriptively without inferential comparison or categorization, consistent with the descriptive exploratory design of the study.

## Results

Diameters of the regular circles

At the first puncture site, the vertical and horizontal diameters of the encompassing circle were 1.60 ± 0.06 mm (n = 5) and 1.58 ± 0.07 mm (n = 5), respectively. At the second puncture site, the corresponding vertical and horizontal diameters were 1.31 ± 0.05 mm (n = 5) and 1.31 ± 0.07 mm (n = 5), respectively.

**Figure 5:**
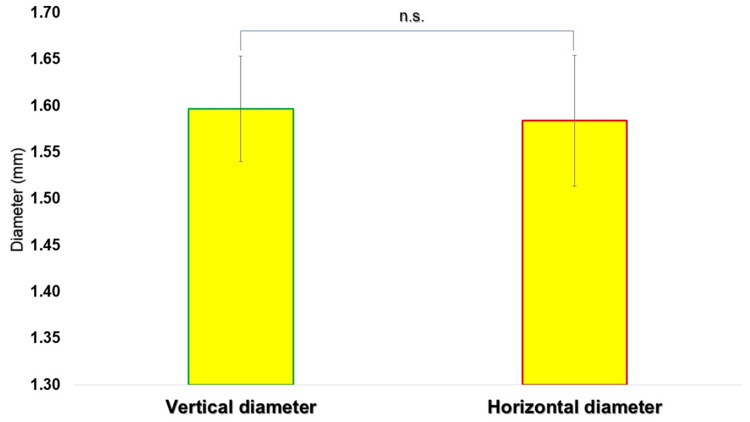
Comparison of the vertical and horizontal diameters (mm) of the perfect circle drawn at the first puncture site The vertical and horizontal diameters were measured from five overlapping images, and the mean and standard deviation were calculated. The vertical diameter was 1.60 ± 0.06 mm (mean ± SD, n = 5), and the horizontal diameter was 1.58 ± 0.07 mm (mean ± SD, n = 5), with no statistically significant difference (p > 0.05). N: number, n.s.: not significant, SD: standard deviation.

**Figure 6:**
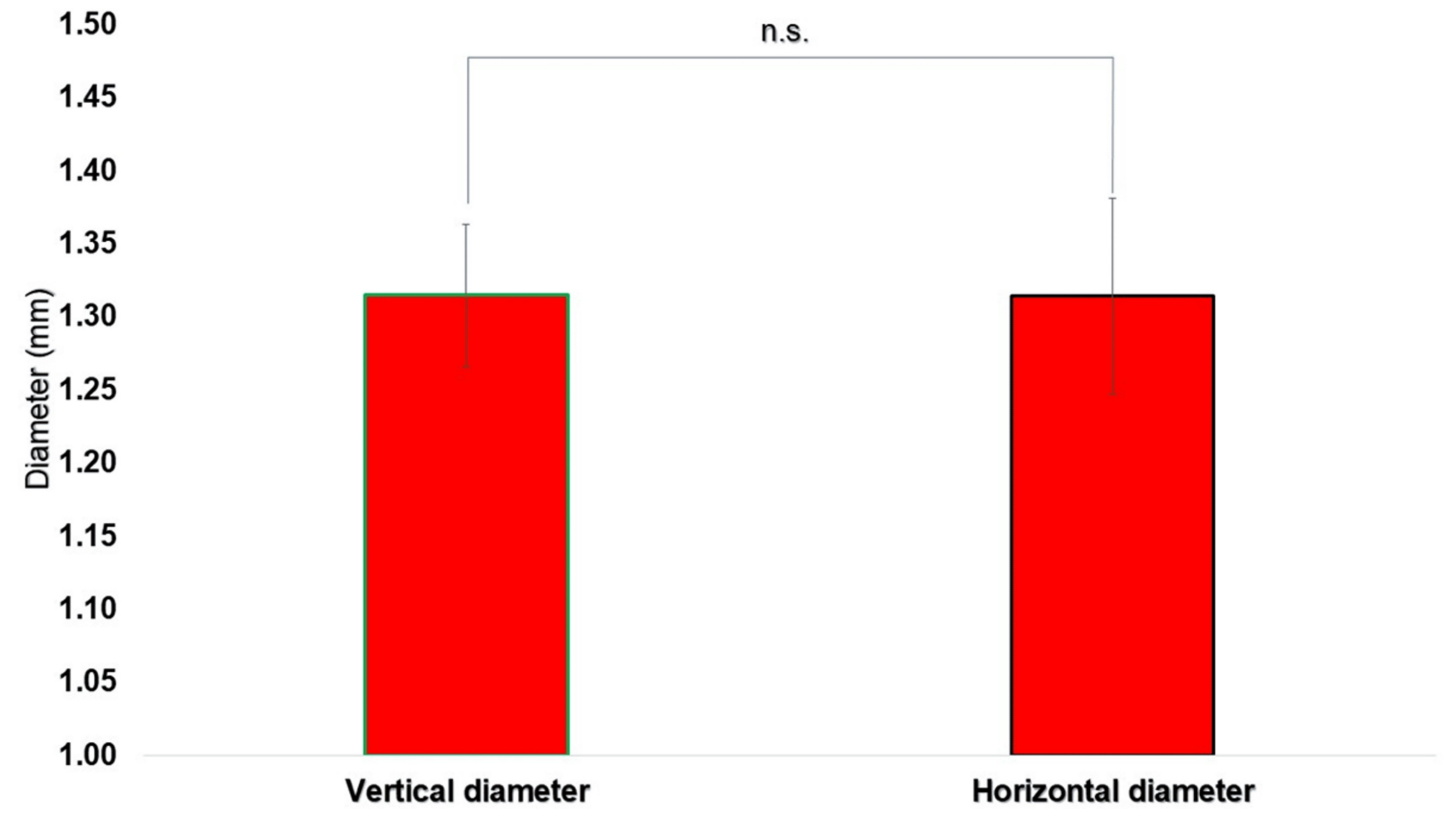
Comparison of the vertical and horizontal diameters (mm) of the perfect circle drawn at the second puncture site The vertical and horizontal diameters were measured from five overlapping images, and the mean and standard deviation were calculated. The vertical diameter was 1.31 ± 0.05 mm (mean ± SD, n = 5), and the horizontal diameter was 1.31 ± 0.07 mm (mean ± SD, n = 5), with no statistically significant difference (p > 0.05). N: number, n.s.: not significant, SD: standard deviation.

No statistically significant differences were identified between the vertical and horizontal diameters at either puncture site (p > 0.05, unpaired t-test) (Figures 5, 6).

**Figure 7:**
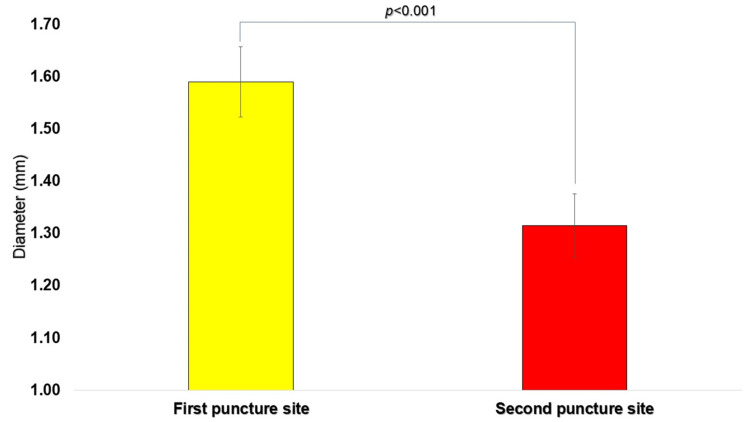
Comparison of the diameter (mm) of the perfect circle drawn at each puncture site Diameters were measured from five overlapping images, and the mean and standard deviation were calculated. Results were 1.59 ± 0.07 mm (mean ± SD, n = 10) for the first puncture site and 1.31 ± 0.06 mm (mean ± SD, n = 10) for the horizontal diameter, demonstrating a statistically significant difference (P < 0.05). N: number; n.s.: not significant; SD: standard deviation.

The mean diameter of the regular circle, calculated as the average of the vertical and horizontal measurements, was 1.59 ± 0.07 mm (n = 10) for the first puncture and 1.31 ± 0.06 mm (n = 10) for the second puncture. The mean diameter was significantly larger at the first puncture site than at the second (p < 0.05, paired t-test) (Figure 7). Homogeneity of variance between the two groups was confirmed by the F-test, with no significant difference observed (p = 0.78).

Count of first-puncture sites included within the second-puncture circle (inclusion rate)

**Figure 8:**
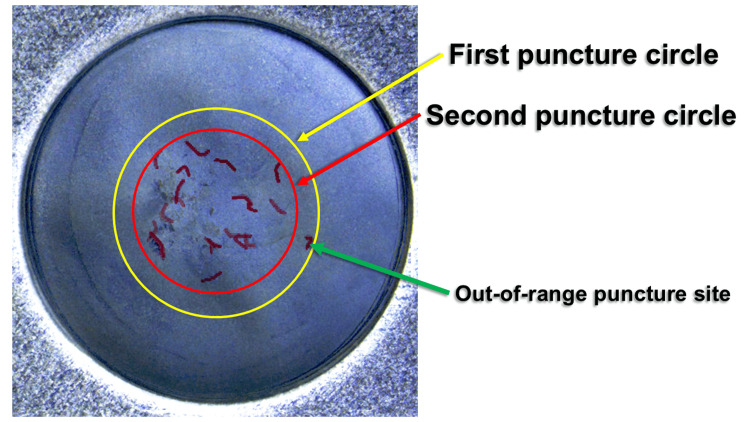
Representative photograph showing that most of the first puncture site is contained within the second puncture circle

The proportion of first puncture sites located within the regular circle delineated by the second puncture site averaged 90 ± 3.2% (n = 5). The majority of first puncture sites were situated within the spatial domain defined by the second puncture site, demonstrating substantial overlap and high reproducibility of puncture positioning (Figure 8).

Minimum distance between the two puncture sites

**Figure 9:**
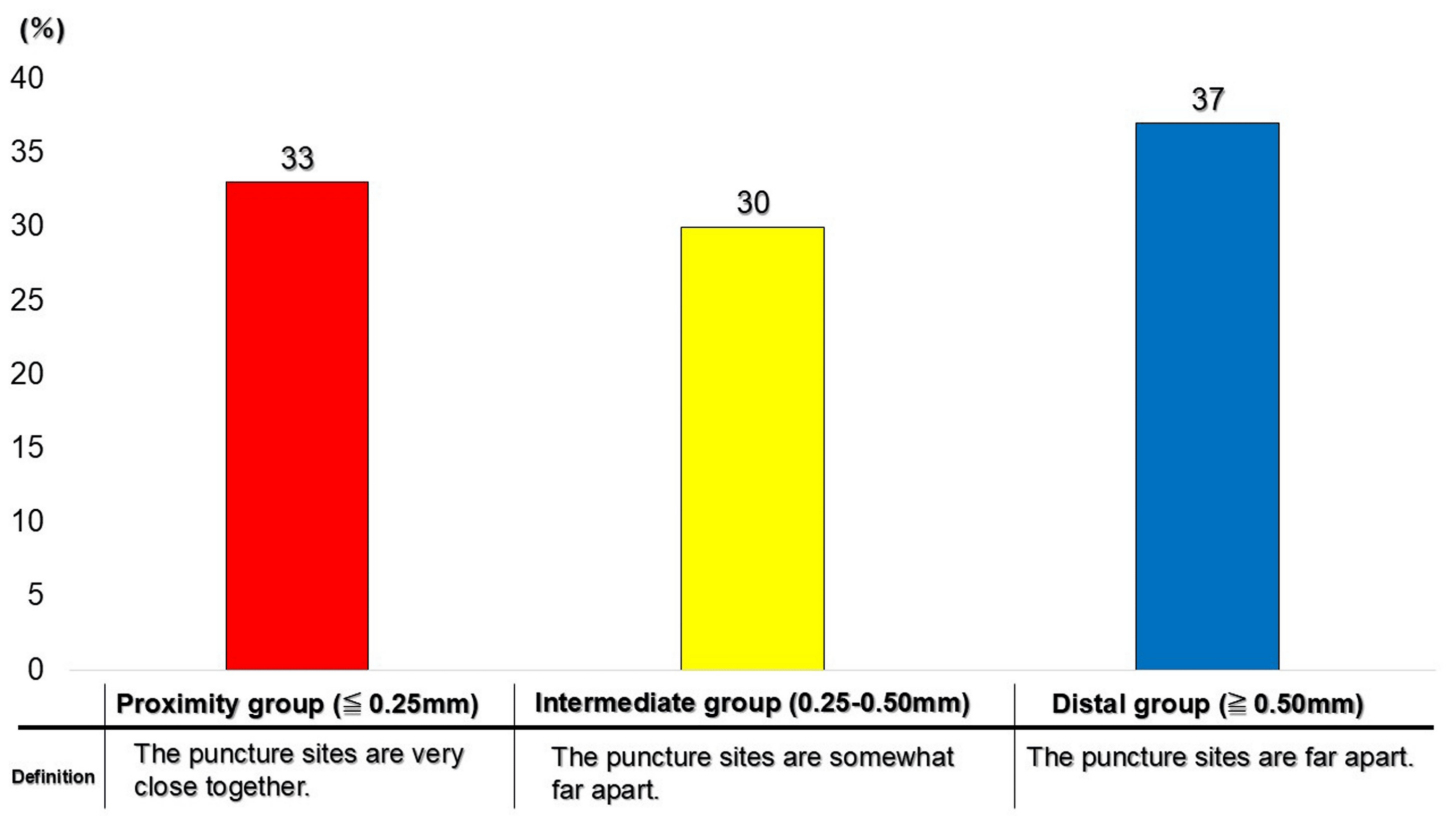
Distribution of the shortest distance between first and second puncture sites

For all 100 cartridges, the minimum linear distance (mm) between the first and second puncture sites was measured as a continuous variable. For descriptive interpretation, the distance distribution was categorized into three ranges: proximity group (≤ 0.25 mm), intermediate group (0.25-0.50 mm), and distal group (≥ 0.50 mm). The resulting distribution comprised 33 cartridges (33%) in the proximity group, 30 cartridges (30%) in the intermediate group, and 37 cartridges (37%) in the distal group (Figure 9). The median (interquartile range) minimum distances were 0.16 mm (0.09-0.19 mm) in the proximity group, 0.35 mm (0.29-0.41 mm) in the intermediate group, and 0.64 mm (0.55-0.75 mm) in the distal group. Overall, one-third of the cartridges demonstrated an inter-puncture separation of ≤ 0.25 mm, indicating extremely close spatial proximity between puncture locations.

## Discussion

This descriptive study quantitatively evaluated the reproducibility and spatial overlap of puncture locations on the rubber stoppers of dental local anesthetic cartridges by performing a second puncture using a standardized procedure. Three spatial indicators, the diameter of the fitted circle, the inclusion rate, and the inter-puncture distance, were used to characterize puncture morphology and spatial displacement.

The diameter of the encompassing circle derived from the first puncture site (1.59 ± 0.07 mm, n = 10) was significantly larger than that of the second puncture site (1.31 ± 0.06 mm, n = 10). The circle diameter observed for the first puncture was consistent with a previously published value (1.50 ± 0.08 mm) [5], supporting the validity of the measurement methodology employed in this study. The larger diameter associated with the first puncture likely reflects variability inherent to clinical manipulation.

In routine clinical practice, a single dental injection needle is commonly used sequentially with multiple cartridges, which may lead to progressive bending or micro-damage of the needle tip. Such subtle deformation can influence the penetration behavior of the needle, potentially altering both the shape and location of the resulting puncture site. Experimental studies of injectable vial systems have reported that repeated puncture can compromise elastomeric stopper integrity, particularly when needle characteristics or insertion parameters vary [10]. Indeed, studies of vial-based injectable products have shown that needle gauge, insertion angle, and needle tip geometry affect both rubber stopper damage and coring incidence [7,11,12].

Furthermore, clinical puncture techniques may deviate from the standardized procedure recommended in package inserts, which typically specify deliberate insertion following cartridge loading. In contrast, clinicians may sometimes advance the cartridge rapidly toward the needle. This difference in technique could increase instantaneous mechanical loading on the rubber stopper and induce a slight deviation of the needle tip, contributing to enlargement of the puncture-associated circular area.

Conversely, the second puncture was performed in strict accordance with the standard procedure and showed a smaller circle diameter. Taken together, these findings indicate that structural differences may arise in puncture morphology between clinically performed punctures and standardized insert-compliant procedures. Few published studies have examined puncture-related stopper damage in dental cartridges using spatial indices, and this analysis therefore offers novel insights.

The inclusion rate averaged 90 ± 3.2% (n = 5), indicating that most first puncture sites were encompassed within the circular region generated by the second puncture. This suggests high reproducibility of puncture positioning when performed under standardized conditions, while also demonstrating a tendency for puncture events to concentrate within a limited spatial region.

Repeated puncture of the same site has been described as a factor contributing to coring [5,7]. Studies of injectable containers have shown that multiple penetrations reduce the self-sealing capacity of stoppers and increase the risk of particle formation [10]. In addition, previous studies have suggested that the occurrence of coring may vary according to the combination of injection needle type and rubber stopper characteristics, highlighting the need for further investigation of material-related factors [13]. The present findings demonstrate that two puncture events frequently occurred within the same localized region, suggesting that double puncture may generate spatial conditions conducive to concentrated mechanical loading on the rubber stopper. However, because this study evaluated spatial characteristics only, it does not establish a direct causal relationship between puncture overlap and coring.

Nevertheless, investigations in other injectable vial systems have demonstrated that rubber coring and particulate generation can occur with measurable frequency when elastomeric stoppers are repeatedly penetrated. Clinical and experimental observations have documented the production of small rubber fragments during routine needle insertion under certain mechanical conditions, supporting the mechanical plausibility that repeated or closely adjacent punctures may increase localized mechanical stress on the stopper surface, even when direct coring events are not specifically measured in the present study [14,15].

Measurement of the minimum distance between puncture sites showed that approximately 30% of cartridges exhibited inter-puncture distances of ≤ 0.25 mm. To facilitate visual and clinical interpretation, distance distribution was categorized into three descriptive ranges: proximity (≤ 0.25 mm), intermediate (0.25-0.50 mm), and distal (≥ 0.50 mm). These thresholds were defined in consideration of measurement resolution and the observed range of puncture diameters and should be regarded as pragmatic indicators for organizing spatial characteristics rather than strict clinical cutoffs.

Based on this categorization, 33% of cartridges were included in the proximity group, demonstrating that puncture convergence within a restricted area occurred with non-trivial frequency. When interpreted alongside the inclusion rate, these results suggest that puncture locations do not distribute randomly across the rubber stopper surface but instead exhibit spatial clustering.

To our knowledge, no previous studies have quantitatively characterized the spatial proximity of two puncture sites in dental local anesthetic cartridges. This descriptive analysis, therefore, represents the first report to define the spatial characteristics of double puncture using quantitative indices. Because dental local anesthetic cartridges are primary packaging for injectable pharmaceuticals and rubber stoppers directly contact the drug, puncture-associated stopper damage is relevant not only to instrument handling but also to pharmaceutical safety considerations.

Several limitations must be acknowledged. First, the second puncture was performed under standardized conditions and did not reflect variability in clinical practice, limiting generalizability. Second, only a single cartridge product was evaluated, and variations across manufacturers may influence puncture behavior. Third, morphological changes in dental injection needles due to repeated use were not directly assessed. Finally, this study evaluated spatial features of puncture sites rather than coring itself, and the relationship between spatial overlap and coring remains to be established. All experiments were performed under in vitro conditions and therefore may not fully represent clinical environments.

## Conclusions

Double puncture of dental local anesthetic cartridges demonstrated a strong tendency to occur within a restricted spatial area, with approximately 30% of cartridges exhibiting inter-puncture distances of ≤ 0.25 mm. These findings indicate that repeated puncture events are likely to converge spatially rather than distribute randomly across the rubber stopper surface.

When an initial puncture fails due to incomplete penetration or deviation of the needle tip, avoiding repuncture of the same cartridge and replacing it with a new one may represent a reasonable measure to mitigate potential rubber stopper damage and reduce the likelihood of coring. To our knowledge, this study is the first spatially based investigation to evaluate the procedural implications of puncture patterns in dental local anesthetic cartridges, offering novel insight for dental clinical practice and the safety management of injectable pharmaceutical products.
